# Targeting z-Crystallin by aspirin restores the sensitivity to cisplatin in resistant A2780 ovarian cancer cells

**DOI:** 10.3389/fphar.2024.1377028

**Published:** 2024-07-03

**Authors:** Matteo Lulli, Andrea Trabocchi, Giandomenico Roviello, Martina Catalano, Laura Papucci, Astrid Parenti, Alice Molli, Cristina Napoli, Ida Landini, Nicola Schiavone, Andrea Lapucci

**Affiliations:** ^1^ Department of Experimental and Clinical Biomedical Sciences “Mario Serio”, Section of Experimental Pathology and Oncology, University of Florence, Florence, Italy; ^2^ Department of Chemistry “Ugo Schiff”, University of Florence, Florence, Italy; ^3^ Department of Health Sciences, Section of Clinical Pharmacology and Oncology, University of Florence, Florence, Italy

**Keywords:** ovarian cancer, chemoresistance, pharmacology, acetylsalicylic acid, RNA binding proteins

## Abstract

Ovarian cancer is the deadliest gynaecologic malignancies worldwide. Platinum based chemotherapy is the mainstay treatment for ovarian cancer; however, frequent recurrence and chemoresistance onset in patients with advanced diseases remain a therapeutic challenge. Although mechanisms underlying the development of chemoresistance are still ambiguous, the B-cell lymphoma-2 (Bcl-2) family is closely associated with chemoresistance in ovarian cancer. We previously disclosed that Zeta-Crystallin (CryZ) is a post-transcriptional regulator of *Bcl-2* gene expression, by binding to *Bcl-2* mRNA and increasing its half-life. Here, we investigated the role of CryZ as a novel therapeutic target in A2780 ovarian carcinoma cells by modulating the protein activity with acetylsalicylic acid (ASA) to restore chemosensitivity. Molecular docking and fragment-mapping based approach revealed potential interaction of ASA within CryZ protein. Inhibition of CryZ binding activity to *Bcl-2* and *Bcl-xl* mRNA targets by ASA was demonstrated in A375 cells. Cytotoxicity assays were conducted in A2780S and A2780R ovarian cancer cells to evaluate if CryZ binding activity inhibition and *CryZ* silencing were able to reverse cisplatin resistance. ASA-treatment determined a downregulation of *Bcl-2* and *Bcl-xl* mRNA levels in A2780S and A2780R cells. ASA-treatment or *CryZ* silencing were able to increase and restore the chemosensitivity in both sensitive and resistant A2780 ovarian cancer cells, respectively. In this research article we demonstrated that the pharmacological or genetic inhibition of CryZ restores the sensitivity to cisplatin in a model of sensitive or resistant ovarian cancer cells. These findings suggest a new gene-targeted chemotherapeutic approach to restore the cytotoxicity in drug-resistant ovarian cancers and increase the sensitivity in non-resistant cells.

## Background

Ovarian cancer is the deadliest gynaecological tumour. In agreement with the National Comprehensive Cancer Network (NCCN) Guidelines for Ovarian Cancer and with European Society for Medical Oncology (ESMO) and European Society of Gynaecological Oncology (ESGO), the standard of care treatment of ovarian cancer is a full-stage surgery and platinum-based systemic chemotherapy (Morgan et al., 2016; Colombo et al., 2019). However, inherent or acquired resistance is the main cause of treatment failure. Even today, the mechanisms responsible for resistance in ovarian cancer treatment are poorly understood (Cornelison et al., 2017). Regarding acquired resistance, the deregulation of anti-apoptotic genes *Bcl-2* and *Bcl-xL* has been well established (Dai et al., 2023; Karademir and Özgür, 2023). Expression of both genes is regulated by post-transcriptional mechanisms, including the interactions between stabilizing or destabilizing RNA-binding proteins (RBP) and their mRNAs (Cui and Placzek, 2018). In the last few years, several aspects of the post-transcriptional control of *Bcl-2* have been characterized, and it has been demonstrated the existence of a complex regulative network controlled by the interaction of an adenylate-uridylate-rich element (ARE) in the 3′UTR region of *Bcl-2* mRNA, with different ARE-binding proteins (AUBPs) (Lapucci et al., 2002; Donnini et al., 2004). Among the *Bcl–2* AUBPs we identified Zeta-Crystallin (CryZ), demonstrating its role in increasing *Bcl-2* mRNA stability in human leukaemia cell lines (Lapucci et al., 2010; Lulli et al., 2020). CryZ exhibits enzymatic activity, particularly acting as NADPH dependent quinone reductase. In the effort to identify pharmacological inhibitors of CryZ, the group of Bazzi MD found that aspirin-like analgesics are potent inhibitors of its enzymatic activity (Bazzi, 2002; Bazzi et al., 2002). Paracetamol (acetaminophen), ibuprofen (2-(4-isobutyl phenyl)-propionic acid), salicylic acid and aspirin (ASA) inhibit the enzyme to varying degrees; the latter being the most potent CryZ inhibitors. The inhibition of CryZ enzymatic activity by ASA occurs through the interaction with a specific binding site, closed to that of NADPH (Trabert et al., 2014; Huang et al., 2016), therefore it appears to be a non-competitive inhibitor with respect to NADPH, interacting with both the free form of CryZ as well as the CryZ/NADPH complex.

The most relevant data on the antitumor role of ASA and other non-steroidal anti-inflammatory drugs (NSAIDs) derive from epidemiologic studies, in a large panel of tumour types (Elwood et al., 2024). Furthermore, a synergistic effect of classical chemotherapeutic agents and ASA on cancer growth and apoptosis has been observed in a human myeloma xenograft model (Mortensen et al., 1998). Also, a reduction in incidence of ovarian cancer has been observed in patients who take ASA regularly (Trabert et al., 2014). In preclinical ovarian cancer models, ASA treatment determined a significant reduction in Bcl-2 protein level with concomitant increase in caspase 3, revealing its antitumor and antiangiogenic effects (Huang et al., 2016). Bcl-2 and Bcl-xl play major roles in the pathobiology and chemoresistance of ovarian cancer, and their inhibitions were useful for treatment (Suh et al., 2023).

In the present research study, we demonstrated that ASA inhibits the binding of CryZ to the mRNAs of the anti-apoptotic *Bcl-2* and *Bcl-xL* genes. In addition, we revealed that modulation of CryZ activity using ASA or its downregulation by specific siRNA increases sensitivity in both sensitive and drug-resistant human ovarian cancer cells in response to cisplatin treatment.

## Materials and methods

### Cell culture

The human A2780S and A2780R ovarian cancer cell lines and the A375 melanoma cell line were purchased from ATCC (ATCC Manassas, United States). A2780S and A2780R cells or A375 cells were maintained in RPMI-1640 or DMEM medium, respectively, supplemented with 10% fetal bovine serum (FBS), penicillin (100 U/mL), and streptomycin (100 U/mL) (Euroclone, Milan, Italy) at 37 C in a humidified incubator with 5% CO2. Cells were periodically screened for *mycoplasma* contamination using PCR.

### RNA-Amply-Seq analysis

Total RNA was isolated from A2780S and A2780R cells by means of NucleoSpin Total RNA kit according to the manufacturer’s protocol (Macherey-Nagel, PA, United States). RNA was quantified by Qubit™ 3.0 Fluorometer (Invitrogen, MA, United States). After quantification, the quality of RNA was evaluated using the Agilent RNA 6000 Nano LabChip® kit with the Agilent 2100 Bioanalyzer (Agilent Technologies, CA, United States). RNA-Sequencing (RNA-Seq) analysis was performed by Novogene (Cambridge, United Kingdom) on an Illumina NovaSeq X Plus system (Illumina, San Diego, CA, United States). Briefly, for each cell line, an experimental triplicate was analyzed. mRNA was purified from total RNA using poly-T oligo-attached magnetic beads. After fragmentation, the first strand cDNA was synthesized using random hexamer primers, followed by the second strand cDNA synthesis using either dUTP for directional library or dTTP for non-directional library. Raw data (raw reads) of fastq format were firstly processed through fastp software. In this step, clean data (clean reads) were obtained by removing reads containing adapter, reads containing ploy-N and low-quality reads from raw data. Paired-end clean reads were aligned to the reference genome (hg38) using Hisat2 v2.0.5. feature Counts v1.5.0-p3 was used to count the reads numbers mapped to each gene and then Fragments Per Kilobase Million (FPKM) of each gene was calculated based on the length of the gene and reads count mapped to this gene.

### Western blot assay

For Western blot analysis, cells were lysed in cold radioimmunoprecipitation assay (RIPA) lysis buffer (1% NP-40, 150 mM NaCl, 5 mM EDTA, 0.25% NaDOC, 50 mM Tris-HCl pH 7.5, 0.1% SDS) supplemented with protease and phosphatase inhibitors (Merck Life Science, Milan, Italy). Total proteins extracted from the cells were quantified with Bradford reagent (Merck Life Science, Milan, Italy). Aliquots of total protein (30 μg) were electrophoresed on 8%–12% (v/v) SDS-PAGE gel (Thermo Fischer Scientific, Milan, Italy), transferred onto nitrocellulose membranes (Bio-Rad, CA, United States) and probed overnight at 4 °C with primary antibodies against CryZ (2A9DD1, Abcam, United States), Bcl-2 (clone 100, Thermo Fisher Scientific), Bcl-xl (SC-8392, Santa Cruz Biotechnologies) and beta-actin (β-actin Thermo Fisher Scientific). Bound primary antibodies were detected using goat anti-mouse or goat anti-rabbit IgG Alexa Fluor 680 or 800 secondary antibodies (LI-COR^®^ Bioscience, Lincoln, NE, United States). Membranes were visualized at the Odyssey Infrared Imaging System (LI-COR^®^ Bioscience, Lincoln, NE, USAEuroclone, Milan, Italy) and densitometric analysis was performed by Odyssey Image studio software (LI-COR^®^ Bioscience, Lincoln, NE, United States Euroclone, Milan, Italy) Quantity One software (Bio-Rad, CA, United States).

### Identification of potential ligand binding sites and hot sposts

A fragment-mapping based approach (FTMAP) was used to locate potential small molecule binding hot spots on each of the Rho crystallographic structures. FTMAP has been shown as a powerful tool for identifying druggable hot spots in proteins (Brenke et al., 2009; Kozakov et al., 2015), as it correlates pocket druggability with propensity to bind clusters of small organic compound fragments.

We used the FTMap method of Vajda et al. to highlight protein surface regions that have the potential to bind the highest number of small molecular probes. The available crystal structure of CryZ (PDB: 1yb5) was subjected to fragment mapping and the resulting hot-spot residues (those that comprise prominent fragment binding sites) were analyzed. Then, complimentary to FTMap studies, we applied FTSite to find likely ligand-binding sites. FTSite uses the hot spots detected by FTMap to identify and rank binding sites, on the basis that the binding site of a protein generally consists of a strong main hot spot plus some other adjacent hot spots (Ngan et al., 2012).

### Docking calculations

Automated docking studies were carried out using the Lamarckian Genetic Algorithm (LGA) as a search engine implemented in the Autodock 4.0.1 program (Morris et al., 1998). The AutoDockTools 1.4.5 (ADT) graphical interface (Gillet et al., 2005) was used to prepare CryZ and ligand PDBQT files. Coordinates of ASA were generated using Spartan (version 5.147), and then energy-minimized through the AM1 semi-empirical method to calculate the equilibrium geometry. The coordinates of CryZ were retrieved from the Protein Data Bank (PDB code: 1yb5), and protein-ligand complex was unmerged for achieving the free protein structure. Water molecules were removed. Hydrogen atoms were added to the enzyme and the ligand, Gasteiger charges were added and non-polar hydrogens were merged. Three-dimensional energy scoring grid of 0.375 Å resolution and 126 Å × 126 Å × 126 Å dimension was computed to include the whole protein. The center of the grid was set on the center of the protein. A total of 50 runs with a maximum of 2500000 energy evaluations were carried out for ASA, using the default parameters for LGA. Low energy ligand-protein complexes were subjected to AMMP energy minimization using VegaZZ (Pedretti et al., 2004), then Cluster analysis was performed on docked results using a root-mean-square (rms) tolerance of 1.5 Å. The analysis of the binding mode of the docked conformations was carried out using the Autodock plugin within PyMol software v0.99 (Alexander et al., 2011).

### Silencing of *CryZ*

The A2780S and A2780R were transfected with 100 nM specific siRNA (IDT, Leuven, Belgium) against human *CryZ* by means of jetPRIME^®^ (Polyplus, Illkirch, France) according to the instruction manual. Scramble siRNA was used as control (si-CTR).

### *In vitro* transcription

*Renilla luciferase* RNA was *in vitro* transcribed to be used as internal RT-qPCR control. A PCR product containing the ORF of Renilla luciferase was obtained from pGL4.71 plasmid, inserting the T7 RNA polymerase consensus by means of PCR reaction. This product was used as a template in the *in vitro* transcription reaction using MEGAscript T7 Transcription kit (Ambion, United States). Obtained RNA was purified using the RNAeasy kit (Qiagen, United States) e quantified by Qubit™ 3.0 Fluorometer.

### RNA immunoprecipitation assay

RNA protein immunoprecipitation (RIP) was performed based on Tenenbaum et al., 2002. In brief, A375 cells were treated with ASA 1 mM the concentration previously demonstrated to inhibit CryZ enzymatic activity in cell free biochemical assays (Bazzi et al., 2002) for 24 h, then cells were lysed in non-denaturing lysis buffer (20 mM Tris-HCl pH 8.0, 137 mM NaCl, 1% Nonidet P-40, 2 mM EDTA) in presence of protease inhibitors cocktail (Merck, United States) and 100 U/mL RNasein ribonuclease inhibitor (Promega, United States). 1 mg of protein extracts were incubated with 15 μL of Dynabeads protein G (ThermoFisher, United States), which were pre-washed with wash buffer (100 mM Tris-HCl pH 7.4, 2 mM EDTA, 150 mM NaCl, 1% Triton X100, 0.2 mM sodium orthovanadate), and with 1 ug of mouse anti-CryZ antibody (2A9DD1, Abcam) or mouse IgG (Santa Cruz, United States) for 18 h at 4°C. Supernatants were then collected, as internal protein control samples, and beads were washed four times using wash buffer. After washing, 30% of beads were used to elute protein for subsequent Western Blotting analysis of immunoprecipitation, and the remaining 70% of beads were subjected to RNA extraction. To this aim, beads were incubated for 15 min at 55 °C in 100 μL of NT2 buffer (50 mM Tris-HCl pH 7.4, 1 mM MgCl_2_, 150 mM NaCl, 0.05% Nonidet P-40) supplemented with 0.5 mg/mL of proteinase K and 0.1% SDS. 1 ng of *in vitro* transcribed *R. luciferase* RNA (as internal control) was added, and total RNA was extracted using RNAqueous total RNA Isolation kit (ThermoFisher, United States). Equal volumes of RNA were retrotranscribed using iScript kit (Biorad). Quantification of *Bcl-2*, *Bcl-xl*, *beta-2 microglobulin* and *R. luciferase* levels was performed through real-time PCR with specific primers (listed in Supplementary Table S1) carried out using SsoAdvancedTM universal SYBR^®^ Green Supermix (Bio-Rad, United States) in a CFX96 Real Time Detection System instrument (Bio-Rad, United States).

### Quantitative real-time PCR

Total RNA was isolated from A2780S and A2780R cells by means of NucleoSpin Total RNA kit according to the manufacturer’s protocol (Macherey-Nagel). RNA was quantified by Qubit™ 3.0 Fluorometer (Invitrogen). 500 ng of total RNA were retrotranscribed using iScript kit (Biorad). Quantification of *Bcl-2*, *Bcl-xl* and *beta-2 microglobulin* was performed through real-time PCR with specific primers (listed in Supplementary Table S1) carried out using SsoAdvancedTM universal SYBR^®^ Green Supermix (Bio-Rad) in a CFX96 Real Time Detection System instrument (Bio-Rad). mRNA was quantified with the ΔΔCt method. mRNA levels were normalized to *beta-2 microglobulin* as an endogenous control.

### Cytotoxicity and drug resistance reversal assays

The growth inhibitory effects of ASA and *CryZ*-siRNA in combination with cisplatin (CDDP) were evaluated in A2780S and A2780R after 72 h treatment by means of the sulforhodamine B (SRB) assay. Briefly, exponentially growing cells were seeded in 96-well plates in complete RPMI 1640 at a plating density of 4 × 10^3^ cells/well. After 24 h, cells were exposed to ASA at 1 and 2.5 µM (concentration doses comparable to those *in vivo* (Dovizio et al., 2012), and previously shown not affecting A2780 cell viability (Huang et al., 2016)) with an increase ranging of concentrations from 1 nM to 100 μM of CDDP or *CryZ*-siRNA 100 nM plus CDDP at the same concentration described above. Each concentration was tested in triplicate. In all cases, after 48-h of drugs or siRNA-drug exposure, cells were fixed with 10% trichloroacetic acid and stained with 0.4% SRB in 1% acetic acid. The SRB fixed to the cells was dissolved in 10 mM Tris-HCl and the absorbance was read at 540 nm on Victor X5 (Perkin Elmer). IC50 values were calculated using GraphPad Prism v7 software (GraphPad Prism La Jolla, CA, United States). The IC50 drug concentration resulting in a 50% reduction in the net protein content (as measured by SRB staining) in drug treated cells as compared to untreated control cells was determined. All the reported IC50 values represent the mean ± SD of three independent experiments. One-way ANOVA test was performed to analyze data.

### Statistical analysis

t-Test and one-way ANOVA test were used for statistical comparisons of samples. *p* values <0.05 were considered statistically significant. All data were analyzed using GraphPad Prism v7 software (GraphPad Prism La Jolla, CA, United States).

## Results

### Bcl-2, Bcl-xl and CryZ expression levels are higher in resistant-compared to sensitive-A2780 cells

**FIGURE 1:**
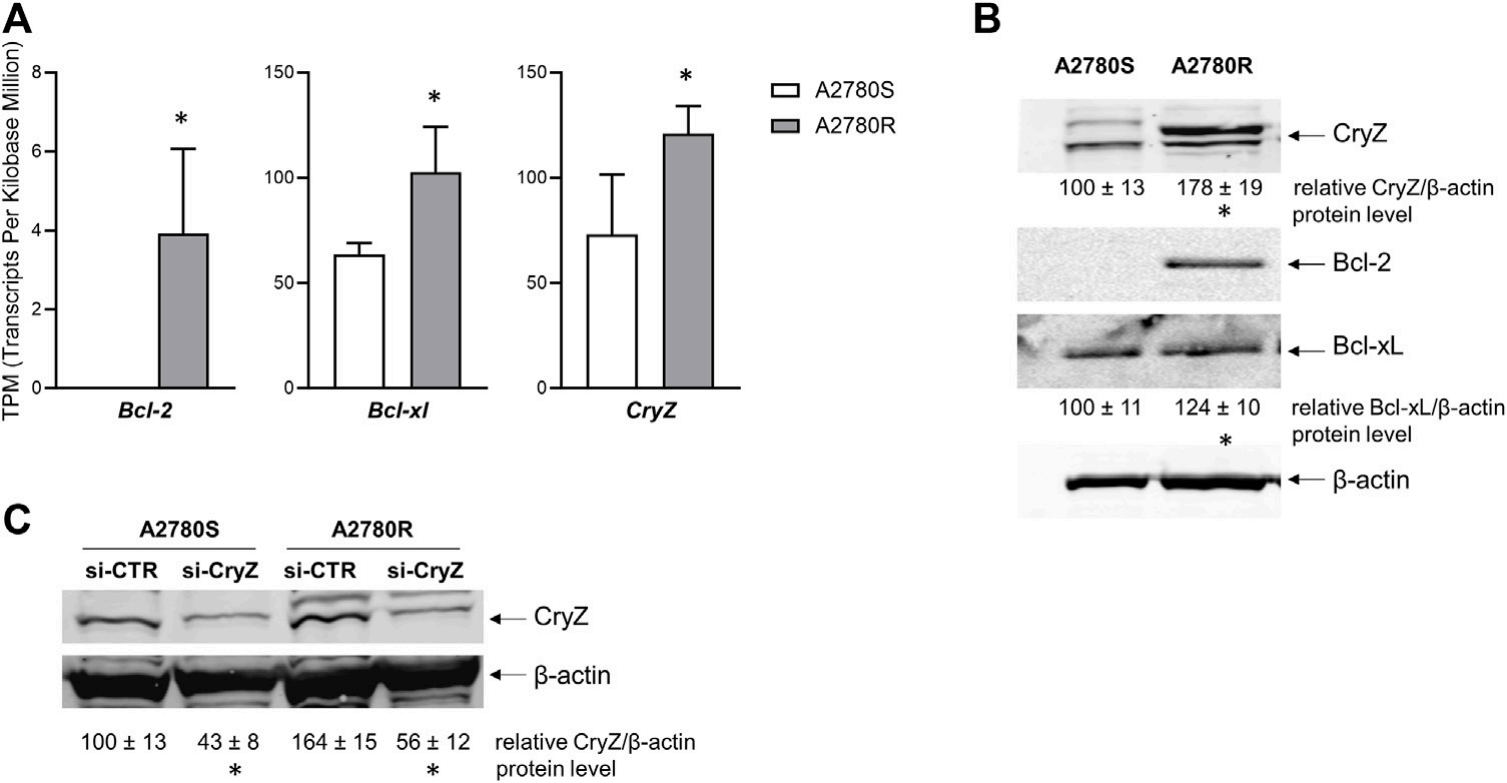
**(A)** Transcriptomic analysis of *Bcl-2*, *Bcl-xl* and *CryZ* genes in A2780S and A2780R; data represent mean ± SD of at three independent experiments (*p < 0.05 vs. A2780S). **(B)** Western Blotting analysis of Bcl-2, Bcl-xl and CryZ, including quantitative analysis, in A2780S and A2780R; the image is representative of three different experiments. β-actin was used as housekeeping control (*p < 0.05 vs. A2780S). **(C)** Western Blotting analysis of CryZ, including quantitative analysis, in A2780S and A2780R transfected with si-CTR or si-CryZ; the image is representative of three different experiments. β-actin was used as housekeeping control (*p < 0.05 vs. si-CTR).

We analysed the expression levels of *Bcl-2*, *Bcl-xl* and *CryZ* genes in the A2780 sensitive and A2780 resistant cells (A2780S and A2780R, respectively) at both RNA and protein levels. As shown in Figure 1A, analysis of transcriptomic data reveals that the A2780R cells show higher expression levels of the three genes compared to the sensitive cells. In agreement with mRNA expression levels, Western Blotting analysis revealed that while in sensitive cells the Bcl-2 protein is apparently absent, its levels result dramatically increased in resistant cells (Figure 1B). In addition, Bcl-xl and CryZ protein levels are higher in A2780R compared to A2780S cells (Figure 1B).

### Identification of interaction site of ASA within CryZ

To elucidate a possible interaction site of ASA within CryZ, two computational methods using molecular docking and fragment-mapping based approach were considered, taking advantage of the available crystal structure of the complex formed by CryZ with NADPH (PDB code: 1yb5).

**FIGURE 2:**
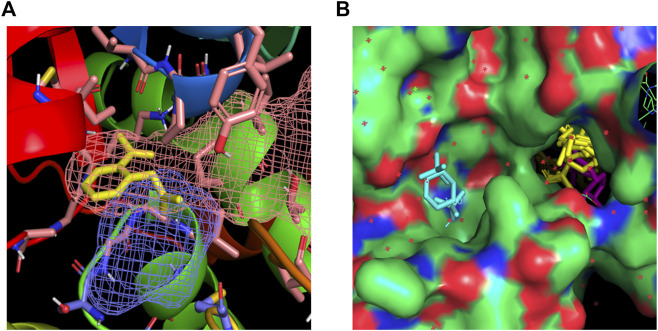
**(A)** Best-binding pose of ASA found in the proximity of the side-chains of Lys324, Asn48, Met325, Gly160 for polar contacts and of Pro49, Gly159, Ile315, Ala321 and Met325 for hydrophobic interactions. The putative binding site found by FTMap and FTSite evaluations are highlighted in orange and blue mesh and in agreement with docking results. **(B)** Fragments populating the hotspots identified within the NADPH (magenta) binding region are highlighted in yellow; fragments in cyan are found within an additional hotspot.

The docking algorithm Autodock 4.0 was used to evaluate the binding energies of minimum-energy conformations of ASA ligand within the whole protein using the largest 3D energy scoring grid available. Docking calculations resulted in a main cluster of 21 out of 50 conformations including the lowest energy one (Supplementary Table S2). All the conformations of the main cluster, including the best docked pose were found within the NADPH binding site, experiencing with the carboxylic group a key salt bridge with Lys324 and hydrogen bonding interactions with Asn48, Met325 and Gly160. The carbonyl oxygen atoms were found establishing a hydrogen bond with NH Val161. As regarding to additional hydrophobic interactions, the best-binding pose of ASA was found in the proximity of the side-chains of Pro49, Gly159, Ile315, Ala321 and Met325 (Figure 2A).

FTMap analysis resulted in the identification of six potential hotspots, five of which were found within two regions in the NADPH binding site, specifically in the phosphodiester and nicotinamide sites (Figure 2B). Docking calculation of ASA within the hotspot not found in the NADPH binding region was carried out, too, resulting in ten-fold lower estimated inhibition constant, as a consequence of modest interaction of ASA within this hotspot (best docked pose was found possessing an estimated free energy of binding of −4.1 kcal/mol, results not shown).

The hotspots identified by FTMap were successively computed by FTSite to identify and rank binding sites. The results confirmed the FTMap evaluation and the docking calculation, identifying a binding site within the NADPH binding region. Figure 2A shows the identified binding site highlighted in mesh, along with the key interacting residues that correspond to those found interacting with ASA in the docking calculation.

Such complementary approaches demonstrated ASA interacting within the nicotinamide site of the NADPH binding region of CryZ, that was found by FTMap and FTSite as a strong main hot spot.

### ASA impairs the binding of CryZ to *Bcl-2* and *Bcl-xl* mRNAs

**FIGURE 3:**
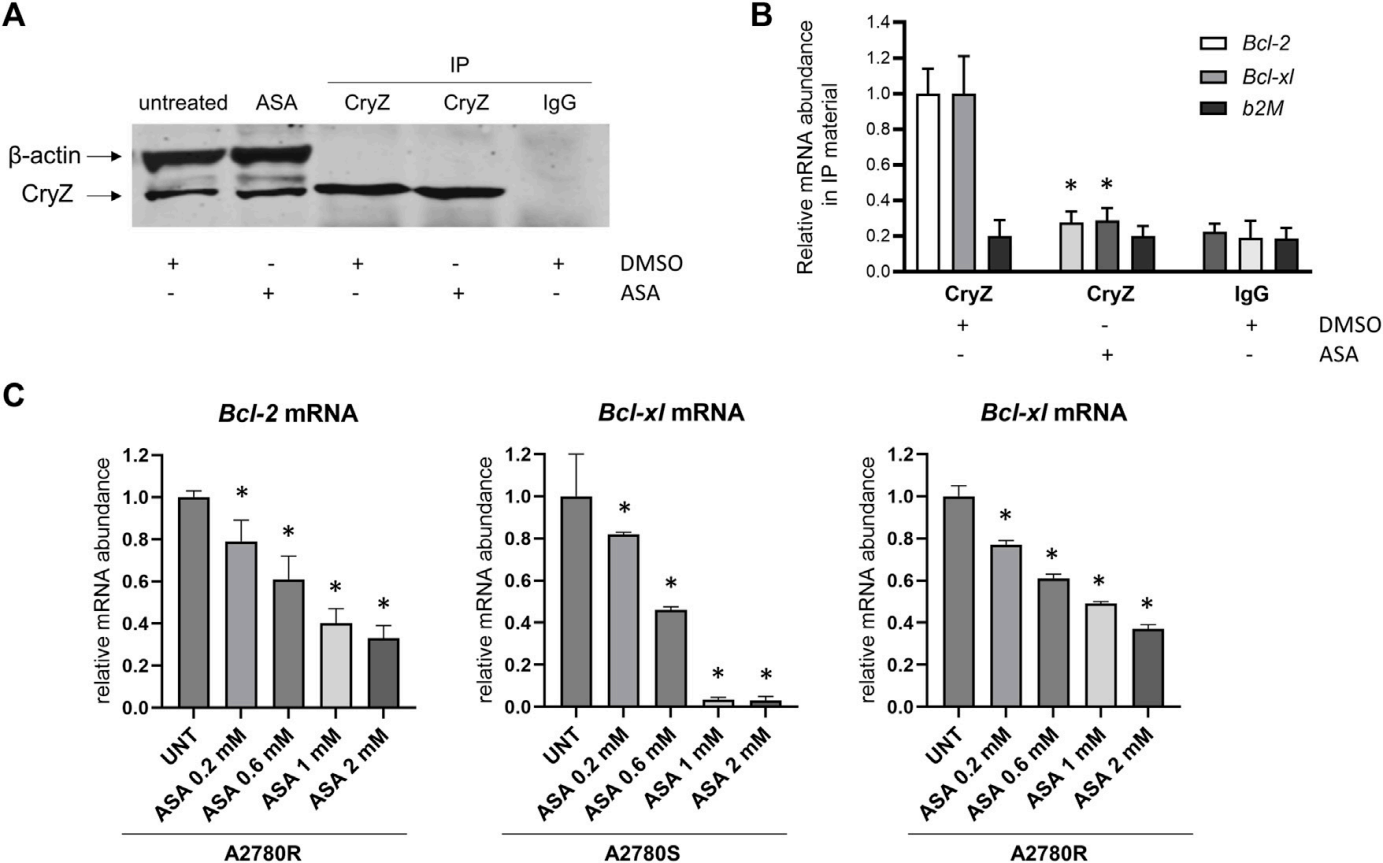
**(A)** Immunoprecipitation assay of CryZ in extracts of A375 cells treated with ASA or with the DMSO alone; β-actin was used as control. **(B)** Evaluation of *Bcl-2*, *Bcl-xl* and *b2M* mRNA expression in immunoprecipitated CryZ-RNA complexes by RNA-immunoprecipitation assay. Statistically significant differences from the control detected by one-way ANOVA are shown (*: *p* < 0.05). **(C)** Evaluation of *Bcl-2* and *Bcl-xl* mRNA expression in A2780S and A2780R cells following ASA treatment (ranging from 0,2 to 2 mM) for 72 h *beta-2 microglobulin* has been used as housekeeping gene. Statistically significant differences from the control (UNT) detected by one-way ANOVA are shown (*: *p* < 0.05).

As shown in Figures 3A,B, RNA-immunoprecipitation experiments carried out on the A375 cells using the anti-CryZ antibody or unrelated IgG as control demonstrate that treatment with 1 mM ASA for 24 h drastically decreased the amount of *Bcl-2* and *Bcl-xL* mRNAs bound by CryZ, but not *b2-microglobulin* used as negative control. This data demonstrates that the presence of ASA impairs the binding of CryZ to these mRNAs, probably by interfering with the binding domain of CryZ to the AU-rich regions. In addition, a 72 h treatment of A2780S and A2780R cells with ASA at different concentrations (from 0,2 to 2 mM) determined a significant dose-dependent reduction of *Bcl-2* and *Bcl-xl* mRNA levels (Figure 3C). Based on the evidence that CryZ is a stabilizing post-transcriptional regulator, this result suggests an increase in destabilization of the two mRNAs following ASA treatment, which could affect drug sensitivity.

### ASA restores the chemosensitivity to CDDP chemotherapeutics in A2780R cells

Table 1 shows how treatment with ASA (1 and 2.5 µM) together with CDDP at different doses for 72 h in A2780S and A2780R cells significantly decreases the apoptotic threshold, increasing sensitivity to CDDP. To highlight the fact that CryZ may be at the basis of the reversion of the resistant phenotype, we performed cytotoxicity experiments following *CryZ* silencing (Figure 1C), treating A2780S and A2780R cells with CDDP at different doses for 48 h. As reported in Table 1, *CryZ* silencing in the A2780R and A2780S cells determined an induction of drug sensitivity, highlighting that CryZ has a major role in drug resistance. Furthermore, these experiments highlight that the inhibition of CryZ binding on the *Bcl-2* and *Bcl-xl* mRNAs by ASA has a fundamental relevance in increasing sensitivity in the A2780S cell line and in reversing resistance in the A2780R cell line.

**TABLE 1:** Cytotoxicity (IC50).

IC50 µM	A2780S	A2780R
CDDP	2.55 ± 0.21	11.16 ± 0.18
CDDP+1 µM ASA	1.41 ± 0.15^a^	9.31 ± 0.21^a^
CDDP+2.5 µM ASA	1.31 ± 0.14^a^	6.32 ± 0.35^a^
siRNA CryZ + CDDP	1.57 ± 0.23^a^	7.34 ± 0.31^a^

All the reported IC50 values represent the mean ± SD, of three independent experiments.

^a^ *p* < 0.05 vs. CDDP (one-way ANOVA, test).

## Discussion

The onset of resistance to chemotherapy drugs is the main determinant of the failure of anticancer chemotherapy in tumours initially sensitive to the drugs (Holohan et al., 2013). Human cell lines resistant to anticancer drugs represent an *in vitro* useful model for studying the mechanisms of action of drugs and the development of tumour resistance. Here, we show that ASA inhibits the binding of CryZ to the mRNAs of *Bcl-2* and *Bcl-xl*, two important anti apoptotic genes and that it is able to revert the drug resistance in ovarian cancer cells. We previously demonstrated that CryZ is a stabilizing RBP of *Bcl-2* (Lapucci et al., 2010), while this is the first demonstration that CryZ is a new RBP of *Bcl-xl*. The mRNAs of both antiapoptotic genes are physiologically able to bind AUBPs by AU-rich sequence element in the 3′-UTR of their mRNAs, thus modulating the recruitment of the degradation machinery constituted by the exosome complex. Therefore, we demonstrated that CryZ could act as *Bcl-2* and *Bcl-xl* AUBP in A2780 ovarian cancer cells. It was previously demonstrated that ASA is a non-competitive inhibitor of CryZ quinone oxidoreductases activity through the interaction with a binding site closed to that of NADPH (Trabert et al., 2014; Huang et al., 2016). Furthermore, Porte et al. demonstrated that NADPH competitively prevents binding of CryZ to synthetic AU-rich RNA sequences, suggesting that the NADPH binding site is involved in CryZ binding to RNA (Porté et al., 2009). Here, by molecular docking and fragment-mapping based approach, we disclose that ASA could interact with CryZ, particularly identifying a binding site within the NADPH binding region. Moreover, we demonstrated that treatment with ASA determines a significant decrease of *Bcl-2* and *Bcl-xl* mRNA expression levels in A2780 cells. In addition, ASA remarkably reduces the ability of CryZ to bind to the target mRNAs, thus it could decrease their stability and ultimately lead to a reversal of the resistant phenotype. This is the first demonstration in ovarian cancer cell lines that chemoresistance can be reverted by the modulation of the post-transcriptional mRNA regulatory pathway.

ASA is the pivotal non-steroidal anti-inflammatory drug (NSAID). To date, NSAIDs are one of the best explored candidate as repurposed drugs for cancer prevention and treatment. In addition to ASA, several commercial NSAIDs, such as ibuprofen, diclofenac, celecoxib and indomethacin, have been qualified as potential antitumor drugs, and some of them are in clinical trials for cancer treatment (Sousa et al., 2023). Furthermore, conjugations of different NSAIDs ligands with platinum-based drugs (such as cisplatin- and oxaliplatin based platinum(IV) complexes) has been recently used to overcome platinum-resistance of ovarian cancer cells (Barth et al., 2023).

We are conscious of the limits of this brief communication in that ASA has a number of other effects that can affect drug sensitivity. However, the fact that *CryZ* silencing mimics the ASA effect at a similar extent suggests that inhibition of CryZ mRNA binding by means of ASA may play a major role. Further studies are needed to finely address mechanistic issues. Considering that the increase of apoptosis resistance is a major player in chemoresistance of ovarian cancer, the impact of molecular characterization of this phenomenon and the identification of putative molecular druggable-targets is of pivotal importance for clinicians. Accordingly, our results could lay the basis for the design of innovative therapies based on CryZ pharmacological inhibition in combination with approved chemotherapies, with the benefit of increasing overall drug effectiveness and reducing side effects.

## Data Availability

The raw data supporting the conclusion of this article will be made available by the authors, without undue reservation. RNA-seq data are deposited at NCBI GEO data repository (GEO dataset GSE270030).
