# Supplementary material for: Targeting z-Crystallin by aspirin restores the sensitivity to cisplatin in resistant A2780 ovarian cancer cells
Source: Front Pharmacol. 2024 Jul 3;15:1377028. doi: 10.3389/fphar.2024.1377028 (PMC11252033; doi:10.3389/fphar.2024.1377028)
Supplement: Supplementary file 1 [file DataSheet1.docx]

**Supplementary material**

**Table S1**

**Primers list**

| Genes | Forward | Reverse |
| --- | --- | --- |
| *CryZ* | CCCGTGGAGACATACATTCGCTCTG | GTAAACAGTGTGGTCTGCTGCAAGAGC |
| *Bcl-2* | GAGGATTGTGGCCTTCTTTGAGTTCG | GAAATCAAACAGAGGCCGCATGCTGG |
| *Bcl-xl* | CAGGCGACGAGTTTGAACTGCGG | CATCTCCTTGTCTACGCTTTCCACG |
| *Beta-2 Micro-globulin* | AGTATGCCTGCCGTGTGAAC | GCGGCATCTTCACAAACCTCCA |
| *Renilla luciferase* | GTCGAGACCATGCTCCCAAGCA | TTGCGGACAATCTGGACGACGT |

**Supporting information S2**

- Cluster analysis
- Coordinates of lowest energy docked conformation

CLUSTER ANALYSIS OF CONFORMATIONS

_________________________________

Number of conformations = 50

Number of distinct conformational clusters found = 12, out of 50 runs,

Using an rmsd-tolerance of 2.0 A

CLUSTERING HISTOGRAM

____________________

________________________________________________________________________________

| | | | |

Clus | Lowest | Run | Mean | Num | Histogram

-ter | Binding | | Binding | in |

Rank | Energy | | Energy | Clus| 5 10 15 20 25 30 35

_____|___________|_____|___________|_____|____:____|____:____|____:____|____:___

1 | -5.72 | 26 | -5.43 | 21 |#####################

2 | -5.55 | 9 | -5.44 | 3 |###

3 | -5.43 | 17 | -5.21 | 4 |####

4 | -5.42 | 46 | -5.24 | 4 |####

5 | -5.27 | 35 | -5.12 | 6 |######

6 | -5.01 | 7 | -4.97 | 2 |##

7 | -4.92 | 1 | -4.92 | 1 |#

8 | -4.69 | 40 | -4.45 | 2 |##

9 | -4.64 | 6 | -4.28 | 2 |##

10 | -4.61 | 27 | -4.61 | 1 |#

11 | -4.46 | 31 | -4.36 | 3 |###

12 | -4.07 | 19 | -4.07 | 1 |#

_____|___________|_____|___________|_____|__________________________________

LOWEST ENERGY DOCKED CONFORMATION

MODEL 26

USER Run = 26

USER Cluster Rank = 1

USER Number of conformations in this cluster = 21

USER

USER RMSD from reference structure = 41.755 A

USER

USER Estimated Free Energy of Binding = -5.72 kcal/mol [=(1)+(2)+(3)-(4)]

USER Estimated Inhibition Constant, Ki = 64.37 uM (micromolar) [Temperature = 298.15 K]

USER

USER (1) Final Intermolecular Energy = -6.55 kcal/mol

USER vdW + Hbond + desolv Energy = -5.00 kcal/mol

USER Electrostatic Energy = -1.56 kcal/mol

USER (2) Final Total Internal Energy = +0.01 kcal/mol

USER (3) Torsional Free Energy = +0.82 kcal/mol

USER (4) Unbound System's Energy = +0.00 kcal/mol

USER x y z vdW Elec q RMS

ATOM 1 C UNK 0001 3.578 -31.289 32.552 -0.43 +0.04 +0.074 41.755

ATOM 2 C UNK 0001 5.321 -32.493 30.700 -0.28 +0.00 +0.003 41.755

ATOM 3 C UNK 0001 4.724 -30.620 32.097 -0.30 +0.05 +0.096 41.755

ATOM 4 C UNK 0001 3.330 -32.585 32.074 -0.49 +0.01 +0.021 41.755

ATOM 5 C UNK 0001 4.198 -33.183 31.156 -0.34 +0.00 +0.001 41.755

ATOM 6 C UNK 0001 5.584 -31.207 31.164 -0.28 +0.01 +0.038 41.755

ATOM 7 C UNK 0001 2.644 -30.636 33.506 -0.47 +0.21 +0.212 41.755

ATOM 8 O UNK 0001 2.421 -29.444 33.617 -0.50 -0.99 -0.644 41.755

ATOM 9 O UNK 0001 2.010 -31.523 34.298 -0.45 -0.72 -0.644 41.755

ATOM 10 O UNK 0001 5.031 -29.318 32.503 -0.23 -0.15 -0.278 41.755

ATOM 11 C UNK 0001 5.016 -28.414 31.448 -0.26 +0.13 +0.260 41.755

ATOM 12 C UNK 0001 6.300 -27.641 31.454 -0.23 +0.05 +0.126 41.755

ATOM 13 O UNK 0001 4.106 -28.240 30.646 -0.74 -0.20 -0.265 41.755

TER

ENDMDL
